# Dietary Patterns in Relation to Cardiovascular Disease Incidence and Risk Markers in a Middle-Aged British Male Population: Data from the Caerphilly Prospective Study

**DOI:** 10.3390/nu9010075

**Published:** 2017-01-18

**Authors:** Elly Mertens, Oonagh Markey, Johanna M. Geleijnse, David Ian Givens, Julie A. Lovegrove

**Affiliations:** 1 Division of Human Nutrition, Wageningen University, 6700AA Wageningen, The Netherlands; elly.mertens@wur.nl (E.M.); marianne.geleijnse@wur.nl (J.M.G.); 2 Hugh Sinclair Unit of Human Nutrition, Department of Food and Nutritional Sciences and Institute for Cardiovascular and Metabolic Research (ICMR), University of Reading, Reading RG6 6AP, UK; j.a.lovegrove@reading.ac.uk; 3 School of Sport, Exercise and Health Sciences, Loughborough University, Loughborough LE11 3TU, UK; 4 Centre for Food, Nutrition and Health, Faculty of Life Sciences, University of Reading, Reading RG6 6AP, UK; d.i.givens@reading.ac.uk

**Keywords:** dietary patterns, principal component analysis, cardiovascular incidence, cardiovascular risk markers, Caerphilly Prospective Study (CaPS)

## Abstract

Dietary behaviour is an important modifiable factor in cardiovascular disease (CVD) prevention. The study aimed to identify dietary patterns (DPs) and explore their association with CVD incidence and risk markers. A follow-up of 1838 middle-aged men, aged 47–67 years recruited into the Caerphilly Prospective Cohort Study at phase 2 (1984–1988) was undertaken. Principal component analysis identified three DPs at baseline, which explained 24.8% of the total variance of food intake. DP1, characterised by higher intakes of white bread, butter, lard, chips and sugar-sweetened beverages and lower intake of wholegrain bread, was associated with higher CVD (HR 1.35: 95% CI: 1.10, 1.67) and stroke (HR 1.77; 95% CI: 1.18, 2.63) incidence. DP3, characterised by higher intakes of sweet puddings and biscuits, wholegrain breakfast cereals and dairy (excluding cheese and butter) and lower alcohol intake, was associated with lower CVD (HR 0.76; 95% CI: 0.62, 0.93), coronary heart disease (HR: 0.68; 95% CI: 0.52, 0.90) and stroke (HR: 0.68; 95% CI: 0.47, 0.99) incidence and a beneficial CVD profile at baseline, while DP1 with an unfavourable profile, showed no clear associations after 12 years follow-up. Dietary pattern 2 (DP2), characterised by higher intake of pulses, fish, poultry, processed/red meat, rice, pasta and vegetables, was not associated with the aforementioned outcomes. These data may provide insight for development of public health initiatives focussing on feasible changes in dietary habits.

## 1. Introduction

Cardiovascular diseases (CVD) are a major public health challenge worldwide, causing substantial morbidity and mortality [1]. Healthy lifestyle factors (including not smoking, an acceptable body weight, high intake of fruits and vegetables, regular physical activity and low or moderate alcohol consumption) are of crucial importance in chronic CVD risk prevention [2]. In addition to promoting the intake of fruits and vegetables, cardio-protective guidelines also support the intake of an overall healthy diet [3]. 

In recent decades, nutritional epidemiology has suggested benefits from assessing the impact of dietary patterns on disease risk instead of a single nutrient and/or food group, as dietary patterns reflect the actual dietary behaviour in the population and thus provide a more comprehensive understanding of how dietary factors together affect the risk of disease [4,5,6]. Dietary patterns can be empirically derived based on the actual dietary habits in a population, with principal component analysis (PCA) as a commonly used *post-hoc* analysis method. Meta-analyses based on prospective studies have indicated that dietary patterns characterised by vegetables, fruits, wholegrains, fish and low-fat dairy products were associated with a decreased CVD risk in the general population, while dietary patterns characterised by red and processed meat, sugar-sweetened foods and drinks, and fried foods were generally associated with an increased risk, but evidence is still limited and inconsistent [7]. 

Only a few epidemiological studies have also investigated the association between dietary patterns and traditional and novel CVD risk markers (including blood pressure (BP), blood lipid levels and inflammation) in a western adult population, but have reported inconsistent results [8,9,10]. Using a cross-sectional design, the ATTICA study among 3042 men and women from Greece revealed that dietary patterns including cereals, small fish, crackers, fruits and vegetables, as well as olive oil in daily cooking and meals were related to a beneficial CVD risk profile at baseline, whereas dietary patterns including sweets, red meat, margarine, nuts with salt and cheese were related to an unfavourable risk profile [11]. Further research is needed to gain insight into the long-term association between dietary patterns and CVD risk markers and events.

The present study aimed to explore dietary patterns, as derived from PCA, in a middle-aged British male population recruited to the Caerphilly Prospective Study (CaPS), and to investigate the association with CVD incidence and, traditional and novel risk markers in a cross-sectional and longitudinal manner.

## 2. Materials and Methods 

### 2.1. Study Design and Study Population

**Figure 1:**
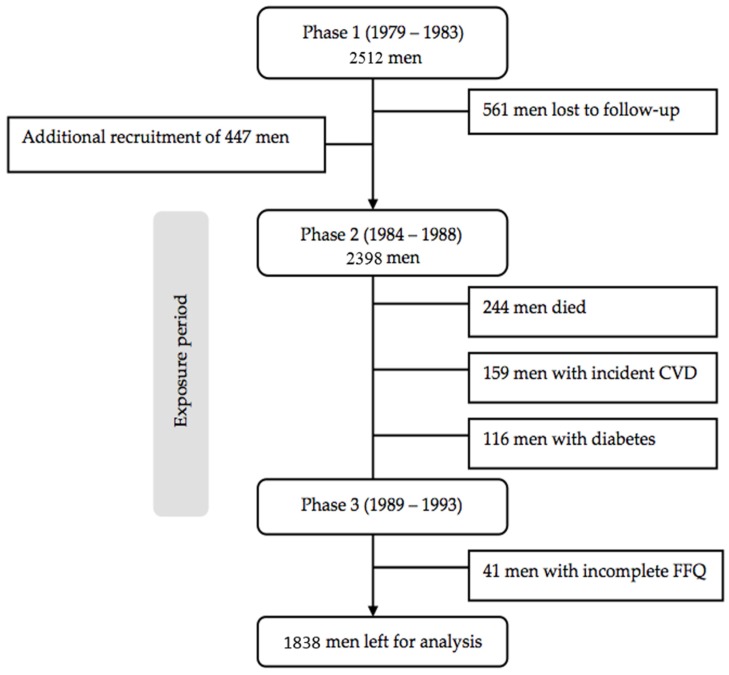
Flowchart of participants through the study.

The CaPS was set up to study the influence of CVD risk factors in the development of coronary heart disease (CHD) [12], and recruited an initial 2512 men, aged 45 to 59 years living in the town of Caerphilly and five adjacent villages, South Wales, UK (response rate 89%). Data-collection phases were at 5-year intervals: 1979–1983 (phase 1), 1984–1988 (phase 2), 1989–1993 (phase 3), 1993–1997 (phase 4), and 2002–2005 (phase 5). At phase 2, an additional 447 men, aged 50 to 64 years, were included as a result of 561 men being lost to follow-up, which gave a new total of 2398 men for the entire cohort. As a result of this change to the cohort, it was deemed necessary to consider phase 2 as baseline in the present study. Before phase 3 measurements, 244 men who died, 159 men who had history of myocardial infarction or stroke and 116 men who had diabetes were excluded from the analyses. After excluding 41 men with incomplete dietary intake data at phase 2 or phase 3, 1838 men were included in the analyses. A flowchart of participants through the study is outlined in Figure 1. Written informed consent was obtained from all subjects included in the study, and the study was approved by the local research ethics committee and adhered to the Declaration of Helsinki.

### 2.2. Exposure Assessment

At phase 2 and phase 3, a semi-quantitative food frequency questionnaire (FFQ) was completed by the subjects, which included estimation of the mean daily consumptions of 50 food items typical for the British diet. Results on the validation study of the FFQ have previously been described in detail [13,14]. Briefly, validity of the FFQ was assessed using a 7-day weighed dietary intake in a representative sample of 665 men (30%), and indicated a statistically significant correlation between methods for all food items ranging from 0.3 to 0.4 (alcohol: 0.75) [14,15]. In the present study, mean dietary intakes over the exposure-period (i.e., phase 2 and phase 3) were calculated to better allow an accurate estimation of dietary intake over time. 

### 2.3. Covariates

The general questionnaires completed by all subjects included questions on demographics, general health and medical history regarding the presence of chronic diseases and risk factors or risk symptoms for CVD. Smoking habits were characterised based on smoking status (never, former or current smoker) and smoking intensity (time since stopped smoking for former smokers and number of cigarettes per day for current smokers). Social class was characterised based on non-manual (including professional, managerial and non-manual occupations) and manual occupations (including manual, semi-skilled and unskilled occupations) [16]. As described previously, physical activity level was assessed using a detailed questionnaire adapted from the Minnesota LTPA questionnaire [17,18]. Briefly, physical activity was characterised as inactive, moderately inactive, moderately active and active based on estimated energy expenditure in leisure time activities during the preceding 12 months [18]. Alcohol consumption was characterised as non-drinking, moderate drinking (≤20 g ethanol/day) or high drinking (>20 g ethanol/day). Phase 2 missing values for smoking habits (0.3%) and social class (0.2%) were replaced by phase 3 values (or phase 1 values) as an alternative for the phase 2 values. 

### 2.4. Cardiovascular Risk Markers

Weight and height were measured at phase 2 and phase 3 for calculation of body mass index (BMI). Resting systolic BP (SBP) and diastolic BP (DBP) were measured at room temperature in duplicate on the left upper arm while the subject was seated using a Hawkslet random 0 sphygmomanometer at phases 2 and 3 and a validated Omron-705CP at phase 5 [19]. At phase 5, measurements of arterial stiffness, namely augmentation index (AIx) and aortic pulse wave velocity (aPWV), were calculated in duplicate by a single operator using a validated SphygmoCor device [20,21,22]. After subjects were fasted for ≤3 h, the pulse pressure wave at the radial artery was recording using applanation tonometry and aPWV was calculated by applanation of the carotid and femoral arteries [20,21,22]. At phase 2, fasting blood samples were taken for analysis of serum total and HDL cholesterol, triacylglycerol, glucose and high sensitivity C-reactive protein (CRP). LDL cholesterol was calculated using the Friedewald Formula [23]. Phase 3 equivalent measures were available for serum total cholesterol, triacylglycerol and glucose. Phase 5 blood assays were limited to triacylglycerol and CRP. Details of the methods have been reported elsewhere [24]. For the present analyses, mean phase 2 and phase 3 variables were generated for BMI, SBP, DBP, total cholesterol, triacylglycerol and glucose. Hypertension was defined as systolic BP ≥ 140 mmHg and/or diastolic BP ≥ 90 mmHg. Phase 2 measurements were used to estimate 10-year CVD risk based on the Framingham Risk Score (FRS) prediction model for global CVD risk that included age, systolic BP not treated, total and HDL cholesterol and smoking status [25]. 

### 2.5. Verification of Outcome 

Incidence of cardiovascular events was confirmed through primary care records, hospital records and the National Health Service Central Registry that also kept death certificates coded by the 9th revision of the International Classification of Diseases (ICD). Clinical endpoints for the present analyses were CHD, including ischaemic heart diseases, cardiac arrest and sudden death (ICD-9 codes: 410–414, 427.5, 798.1, 798.2, 798.9), stroke (ICD-9 codes: 430–434, 436) and CVD incidence, including both fatal and non-fatal CHD, stroke and congestive heart failure events (ICD-9 codes: 428). 

### 2.6. Statistical Analysis

#### 2.6.1. Identification of Dietary Patterns

The 50 food items included in the FFQ were grouped into 28 food items/groups, which were applied in the PCA to derive dietary patterns [26]. An orthogonal rotation procedure, the varimax, was used to simplify the factor structure and render it more easily interpretable. Three principal components were retained, based on components that had an eigenvalue >1 and a graphical evaluation of the Scree plot of eigenvalues (i.e., the point at which the slope of the plot changes) [27]. In agreement with previous literature [28,29,30], an absolute factor loading >0.20 was used to define food items/groups, which made a meaningful contribution to dietary patterns. Component scores were calculated for each of the three dietary patterns identified. These scores were formed by standardising each variable to a zero mean and standard deviation of one, weighing it with a corresponding component score coefficient, and then summing the terms. Thus, for each subject the component score indicated the extent to which his diet conformed to one of the dietary patterns identified. A high factor score for a given dietary pattern indicated high intake of the food items/groups within that food pattern, and a low score indicated low intake of those food items/groups. For the analyses, subjects were classified into tertiles depending on their component score, using the lowest tertile (lowest component score) as reference. 

#### 2.6.2. Dietary Patterns versus Cardiovascular Events

Hazard ratios (HR) and the 95% confidence intervals (CI) were computed for the association between dietary patterns and CVD, CHD or stroke incidence using Cox regression analyses. Follow-up was defined as the time starting from phase 3 measurements until the onset of CVD event, or censoring (mortality from another cause of death, loss to follow-up or final follow-up assessment in September 2014), whichever came first. Model assumption of the proportional hazard analyses was confirmed by graphical evaluations of log-minus-log plots. Adjustments were made for phase 2 age (continuous), phase 2 smoking status and intensity (categories), phase 2 social class (categories), phase 2 physical activity level (categories), mean phase 2 and phase 3 energy-intake (continuous) and mean phase 2 and phase 3 usual alcohol consumption (categories). 

To study the possible mediating role of various risk markers in the relationship between dietary patterns and CVD events, incremental models included BMI, SBP, DBP, total, LDL and HDL cholesterol, Total:HDL cholesterol, triacylglycerol, glucose and CRP separately in the multivariable model. Also, interaction models including interactions terms between dietary patterns and age (<50 and ≥50 years), smoking status (never, former and current), alcohol consumption (none, moderate and high), BMI (<25 and ≥25 kg/m^2^), hypertension status (yes and no), blood lipids (Total:HDL cholesterol; continuous) or inflammation (CRP; continuous) in the multivariable model, were performed to study a possible effect modification by these factors, and a significant interaction was further studied by stratified analyses.

#### 2.6.3. Dietary Patterns versus Risk Markers 

Furthermore, the association between dietary patterns and CVD risk markers was further explored using linear regression analyses. Both the cross-sectional association with mean phase 2 and phase 3 risk markers and the longitudinal association with phase 5 risk markers were investigated. The longitudinal analyses, representing a follow-up of 11.8 (SD 1.1) years, were based on 760 subjects for whom data on phase 5 measurements were available. Adjustments were made for similar lifestyle factors as stated above, with an additional adjustment for BMI. Model assumptions for linear regression were confirmed by plots of outcome variable versus explanatory variables and normal probability plots, and deviations against normality were repaired using log transformations. 

All analyses were carried out within the statistical software programme STATA, version 14 (STATA Corp, College Station, TX, USA), and a two-sided *p*-value below 0.05 was considered as statistically significant.

## 3. Results

### 3.1. Dietary Patterns 

In the CaPS, three dietary patterns, that explained 24.8% of the variance in the original dietary variables, were identified using PCA; their complete component-loading matrix is presented in Table 1. Dietary pattern 1 (DP1) explained 10.3% of total variance in food intake and was mainly characterised by higher intakes of white bread, butter, lard, chips and sugar-sweetened beverages, processed meat and lower intakes of wholegrain bread. Dietary pattern 2 (DP2) which accounted for 8.4% of total variance was characterised by high intakes of pulses, all kind of meat products (including poultry, processed and red meat), fish products (including white and oily fish), rice, pasta, vegetables, fruits and eggs. Dietary pattern 3 (DP3), which explained 6.1% of total variance, was characterised by high intakes of sweet puddings and biscuits (including digestive biscuits or plain biscuits, sweet biscuits, sweets or jellies, ice cream, sweet yoghurt or chocolate, fruit cake or sponge cake and, fruit tart or jam tart), wholegrain breakfast cereals and cream, followed by milk puddings and milk, but was negatively correlated with alcohol intake. 

### 3.2. Descriptive Statistics

During a mean follow-up of 16.6 (SD 7.2) years, 715 CVD events (501 non-fatal and 214 fatal) were identified in the total cohort of 1838 men, of which 402 were reported as CHD (243 non-fatal and 159 fatal) and 205 as stroke (178 non-fatal and 27 fatal). Elevated levels for major CVD risk markers (including BMI, systolic BP and, total and LDL cholesterol) were observed at phase 2, resulting in a FRS around 27% that implies a high risk for the development of CVD in the next 10 years (Table 2). Characteristics of the subjects according to the tertiles of the dietary patterns are given in Supplementary Materials (Tables S1–S3). Subjects with higher scores on DP1 were more likely to smoke, be employed in a manual occupation and consume lower intakes of dietary fibre, fruit and vegetables (Supplementary Materials Table S1). Higher scores on DP2 were more likely to be associated with a high level of leisure time physical activity and as expected higher intakes for meat, fish, vegetables and fruit (Supplementary Materials Table S2). Subjects with higher scores on DP3 were less likely to smoke or consume alcoholic beverages, and were more likely to be a non-manual worker. Focussing on the 10-year risk estimation, subjects with higher scores on DP1 were more likely to have a high FRS, whereas subjects with higher scores on DP3 were more likely to have a low FRS (Supplementary Materials Table S3). 

### 3.3. Incidence of CVD, CHD and Stroke 

DP1 was significantly associated with a higher risk for CVD (HR: 1.35; 95% CI: 1.10, 1.67) and stroke (HR: 1.73; 95% CI: 1.23, 2.42) when comparing the highest tertile with the lowest after multivariable adjustment (Table 3). No significant association with CHD was observed. DP2 was not associated with CVD when comparing the highest tertile with the lowest (HR: 1.12; 95% CI: 0.93, 1.35). Similarly, DP2 was not related to CHD and stroke outcomes. DP3 was significantly associated with a lower risk of developing CVD, CHD and stroke; HR of 0.76 (95% CI: 0.62, 0.93), 0.68 (95% CI: 0.52, 0.90) and 0.68 (95% CI: 0.47, 0.99) respectively were found for men in the highest tertile of DP3 component scores compared with the lowest after adjusting for potential confounding factors. 

Incremental models, including BMI, systolic and diastolic BP, total, LDL and HDL cholesterol, Total:HDL cholesterol, triacylglycerol, glucose or CRP in the multivariate model did not alter the results, except the DP3-associations were slightly attenuated (data not shown). No significant interactions between dietary patterns and age, smoking status, hypertension, glucose or CRP were observed for CVD risk (data not shown), while BMI interacted with DP1, and alcohol and Total:HDL cholesterol with DP3. In DP1, stratification by BMI showed a stronger increased risk for subjects with a normal BMI (HR: 1.56; 95% CI: 1.02, 2.37). In DP3, stratified by alcohol consumption yielded stronger association for none and high alcohol consumption than for moderate alcohol consumption, with a significant decrease in CVD risk for high alcohol consumption (HR: 0.53; 95% CI: 0.32, 0.88). Stratification by Total:HDL cholesterol resulted in stronger associations for subjects with a ratio below 5 than for a ratio ≥5, showing a significant decrease in CVD risk for ratio <5 (HR: 0.61; 95% CI: 0.42, 0.89).

### 3.4. Cardiovascular Risk Markers

DP1 was negatively associated with baseline BMI, but positively associated with DBP, blood glucose and FRS after multivariable adjustment; comparing the highest tertile with the lowest, BMI was a significant −0.58 kg/m^2^ lower (95% CI: −1.03, −0.13), DBP was a significant 1.57 mmHg higher (95% CI: 0.26, 2.85), blood glucose was a significant 0.17 mmol/L higher (95% CI: 0.08, 0.26), and FRS was a significant 13% higher (95% CI: 6, 21) (Supplementary Materials Table S4). When analysed longitudinally, only CRP was a significant 30% higher (95% CI: 4, 62) for the second tertile compared with the lowest, with no differences observed between the highest and the lowest tertile (Table 4). 

For DP2, when comparing the highest tertile with the lowest, baseline BMI was significant 0.52 kg/m^2^ higher (95% CI: 0.12, 0.92) (Supplementary Materials Table S4) and phase 5 AIx was a significant 1.74% higher (95% CI: 0.02, 3.46) (Table 4). No other associations between DP2 and CVD risk markers were observed.

DP3 showed an inverse association with baseline BMI, SBP, triacylglycerol, CRP and FRS after multivariable adjustment; comparing men in the highest tertile with the lowest, BMI was significantly lower −0.99 kg/m^2^ (95% CI: −1.43, −0.55), SBP was significantly lower −3.07 mmHg (95% CI: −5.50, −0.64), triacylglycerol was significantly lower −8% (95% CI: −32, −4), CRP was significantly lower −15% (95% CI: −27, 3), and FRS was significantly lower −7% (95% CI: 3, 14) (Supplementary Materials Table S4). No clear association with phase 5 risk markers was found for this dietary pattern (Table 4).

## 4. Discussion

In the CaPS of 1838 middle-aged men, DP1, mainly characterised by higher intakes of white bread, butter, lard, chips, sugar-sweetened beverages, processed meat and lower intake of wholegrain bread was associated with higher incidence of CVD and stroke, and unfavourable CVD risk profile at baseline. DP3, mainly characterised by higher intakes of sweet puddings and biscuits, wholegrain breakfast cereals and dairy (excluding cheese and butter) and lower alcohol intake, was associated with a decreased risk of developing CVD, CHD and stroke events and a favourable profile of CVD risk markers at baseline. DP2, mainly characterised by higher intakes of fish, poultry, processed/red meat, rice, pasta, vegetables, fruits and eggs, showed no significant associations with CVD risk. After a 12-year follow-up, no clear associations between dietary patterns and CVD risk markers were found. 

The relationship between dietary patterns and CVD risk has been investigated previously. However, because of differences in dietary pattern composition, partly explained by study population characteristics and time-culturally defined variations in eating habits [31], direct comparisons between studies is difficult. In the present study, DP1, characterised as a generally ‘unhealthy’ dietary pattern, was associated with higher incidence of CVD and stroke. In contrast, a similar ‘high-fat/low fibre’ dietary pattern’ (high in red meat, meat products, white bread, fried potato, eggs) was only associated with an increased risk of all-cause mortality in older male cohort from the British Regional Heart Study [30]. DP1 was associated with a higher baseline diastolic BP, fasting blood glucose and FRS, as well as a higher incidence of CVD and stroke. Similarly, recent literature revealed an increased 10-year CVD risk prediction, as assessed by the FRS, for a dietary pattern which included higher intakes of white grains and soft drinks [32]. We found that DP1 was negatively associated with BMI. This is in agreement with adverse weight profiles not been identified as an established risk factor for stroke [33]. Furthermore, we found that subjects with higher scores on DP1 were more likely to smoke. Cigarette smoking, an established CVD risk factor, is associated with lower BMI [34,35] and may contribute in part to the significant relation between this DP and CVD risk [33].

Previous evidence supports a decrease in the intake of refined grain and an increase in the intake of wholegrain, resulting from the protective health associations of wholegrains on CVD and its associated risk markers [36,37]. As a result of the clearly established link between SFA intake and LDL cholesterol, limiting intake of SFA to ≤10% of total energy intake (%TE) is a major public health dietary recommendation for CVD risk reduction [38,39,40]. In this cohort, the SFA intake was around 18.0%TE among those in the highest tertile compared with 14.6%TE in the lowest tertile and represents a high dietary intake compared with current SFA intakes in UK adults, with an average intake of 12.1%TE [41]. This is in agreement with research which has shown a significant relationship between SFA and CVD events [42,43]. Conversely, some recent meta-analyses of prospective studies have not supported cardiovascular recommendations that encourage low consumption of SFA [44,45]. These discrepancies could be partly attributed to diet-disease relationships being dependent on the foods that replace SFA and possibly the specific food sources of SFA [46]. More specifically, diets high in SFA from butter were consistently associated with increases in plasma total cholesterol and LDL cholesterol in well-controlled randomised trials [47,48] and SFA replacement with unsaturated fats was associated with significant reduction in CHD [42,43]. In the present study, consumption of butter and lard were the main contributors to the high SFA intake, which is in agreement with previous CaPS findings showing that butter consumption was associated with multiple markers of increased CVD risk, in particular BP and vascular stiffness [49]. 

In the context of the literature on dietary patterns, DP2 of the present study can be described as a pattern that is close to a ‘prudent’ dietary pattern, as identified in the Nurses’ Health Study and the Health Professional’s Study [50,51,52,53], but with high protein from all sources including processed and red meat, and a high fruit intake. These deviations might have attenuated the positive associations of the food items/groups of the prudent pattern (i.e., fruit and vegetables, fish, poultry and wholegrains) that have shown beneficial associations with CVD [50]. In contrast, it might also be plausible that the combined effects of beneficial intakes of vegetables and oily fish could have attenuated the expected detrimental associations of high intakes of processed and red meat [54,55,56]. This attenuation has recently been observed in a cross-sectional study that found a reduced 10-year CVD risk prediction, as assessed by FRS, for the prudent dietary pattern with higher intakes of fresh fruit, vegetables, and whole grains, but no association with CVD risk prediction for a dietary pattern with high intakes of meat, eggs, fats, fish and poultry [32]. Our findings are comparable with the British Regional Heart Study, which demonstrated that a “prudent” dietary pattern was not significantly associated with cardiovascular outcomes or mortality in older men [30].

The lower risk of developing CVD, CHD and stroke in those consuming the DP3 are supportive of previous reports for a beneficial relation between high milk and dairy intake with CVD [57] and stoke risk [58]. In the current study, DP3 refers to the intake of dairy products including cream, milk pudding and milk (primarily whole milk [59,60]), but excludes cheese and butter consumption. Previous CaPS results could not provide conclusive evidence for the beneficial association between milk consumption and CHD or stroke [61], this highlights the additional value of assessing the cumulative associations of multiple food groups included in a dietary pattern on CVD risk, rather than restricting associations to one food or nutrient [62]. Interestingly, this study revealed that dairy consumption was related to high intakes of sweet puddings and biscuits, and wholegrain breakfast cereals, of which the latter has previously been associated with health benefits [63]. The former contributing primarily empty calories from fat and added sugars to the diet was associated with higher intakes of total fat (36.7% vs. 34.6%TE) and total sugar (20.0% vs. 17.0%TE) in the diet among those in the highest tertile, compared with those in the lowest tertile. The findings of the present study indicated that consumption of low nutrient, high-energy dense food products can still be part of a dietary pattern associated with lower CVD risk. Additionally, the lower alcohol consumption of around 5 g ethanol/day observed in DP3 might contribute to the beneficial association found for this dietary pattern and is in line with evidence of the “J”-shaped curve for describing the association between alcohol consumption and cardiovascular risk [64].

The associations between DP3 and CVD risk were only slightly attenuated when adjusting for CVD risk markers, implying that the understanding of CVD pathogenesis through its risk markers is still incomplete. Furthermore, similar results were obtained when alcohol was excluded from the model (data not shown). Notwithstanding, the cross-sectional analyses also suggested that the food items/groups included in DP3 might have been associated with CVD risk reductions through different biological pathways, including traditional and novel risk markers, since BMI, SBP, triacylglycerol and CRP at baseline were inversely associated with DP3. This is in agreement with the literature that has shown beneficial associations on cardiovascular health for both dairy [65,66] and wholegrain consumption [37,63]. Previously, dairy consumption has been associated with lower BP [65,67], and wholegrain consumption with lower BP, total and LDL cholesterol, glucose [63] and CRP levels [68]. 

In the present study, three dietary patterns were identified, resulting in different strengths of associations with CVD risk. This indicates that the complex combinations of food intake might play an important role in the development of CVD. Dietary pattern analysis is therefore regarded as a more robust and valid measure of dietary intake in the longer term, since identification of dietary patterns is based on the correlations in dietary habits of the population under study [4,10]. However, the heterogeneous dietary habits of a population might result in numerous minor dietary patterns, and to fully understand the diet-disease relation in the total population further research is required into these specific dietary patterns. 

Strengths of the present study include its prospective design, with multiple measurements over time and its relatively long follow-up period (mean: 16.6 (SD 7.2) years), to investigate the cross-sectional and longitudinal association between diet and both traditional and novel cardiovascular risk markers in a community-based population. However, outcome measures in phase 5 were available for only 40% of the population under study, which resulted in a reduction in study power that may have contributed to the loss of significance in the longitudinal compared to the cross-sectional analyses. Dietary intake was assessed through a self-reported validated FFQ [13,14] administrated at phase 2 and phase 3, allowing a more accurate estimate of long-term dietary intake. However further dietary assessment was not undertaken for the subsequent follow-up period and no account was taken of possible dietary changes during this period, which could be considered a limitation [30]. It is acknowledged that under or over-reporting were not accounted for in the present study and this may have biased the results [69]. However, we did adjust for energy intake in our models. Apart from possible dietary measurement error, the identification of dietary patterns might have been influenced by subjective judgments of the PCA regarding the grouping of food items/groups included for analysis, the selection of components to retain and the component loading chosen to describe the dietary patterns. In contrast to *a priori* approaches, which are hypothesis driven or pre-defined based on dietary guidelines, it is acknowledged that our data driven *a posteriori* approach does not build on existing research findings and that there may be issues surrounding the validity and reproducibility of the data [27,30]. However, when compared to *a priori*-defined dietary patterns, a strength of the latter approach is that it is not reliant on previous hypotheses and provides scope for characterizing the overall diet of a population and can be used for hypothesis generation [30]. Since dietary patterns are generated based on available empirical dietary data, they are specific for the population under study, but do not necessarily represent a healthy dietary pattern, which might partly explain the weaker association observed with CVD risk markers. The three dietary patterns identified in the present study explained only 25% of the total variance, suggesting heterogeneous eating habits of this middle-aged male population and a possible existence of additional minor dietary patterns that were not evaluated. Secondly, the lack of data on dietary intake at phase 5 and medication use are limitations to the study. It is acknowledged that changes to lifestyle e.g., dietary behaviour, as well as the initiation of prescription drugs (including blood pressure medications and statins) prior to follow-up, could provide some explanation for reductions in key risk markers over time. Finally, the results observed could be partly affected by residual confounding despite the adjustment for relevant factors, as with any observational study, and might not be applicable to non-Caerphilly populations. Although there is no discussion of CaPS in relation to ethnicity or race, a review by Ranganathan and Bhopal noted that racial or ethnic minorities are likely to be underrepresented in this cohort, due to the relatively rural location in which the study was based in South Wales [70]. This study is a representative sample of middle-aged men from South Wales and the *a posteriori*-defined dietary pattern findings may not be generalizable across other populations, including women, other age groups, geographical locations and non-white ethnic groups [70,71]. 

In conclusion, in this cohort of middle-aged men, a dietary pattern mainly characterised by white bread, butter, lard and sugar-sweetened beverages and low wholegrain intake was related to a higher risk of developing incident CVD and stroke, whereas a dietary pattern mainly characterised by sweet puddings and biscuits, wholegrain breakfast cereals and dairy (excluding butter and cheese) and low alcohol intake was related to a lower risk of developing CVD, CHD and stroke. The latter pattern was also associated with a favourable CVD risk profile at baseline. The dietary patterns that have been identified could add to the knowledge base for CVD prevention initiatives focusing on more feasible improvements in the dietary habits at a population level.

## Figures and Tables

**Table 1:** Rotated component loadings derived from principal component analysis for dietary variables that constitute the 3 main dietary patterns identified in the CaPS ^1^.

Food Items/Food Groups	Empirically Derived Dietary Patterns ^2^		
	Dietary Pattern ^1^	Dietary Pattern ^2^	Dietary Pattern ^3^
White bread	0.37	−0.04	−0.05
Wholegrain bread	−0.32	0.15	0.03
White breakfast cereals	−0.04	−0.02	0.09
Wholegrain breakfast cereals	−0.20	−0.01	0.36
Red meat	0.21	0.26	0.06
Processed meat	0.20	0.29	−0.17
Poultry	−0.09	0.35	−0.02
White fish	−0.07	0.31	0.03
Oily fish	−0.05	0.33	−0.05
Potatoes	−0.07	0.08	0.25
Chips	0.30	0.10	−0.09
Vegetables	−0.17	0.26	0.19
Pulses	0.03	0.36	−0.05
Pasta	0.02	0.26	0.02
Rice	0.03	0.29	0.06
Sweet puddings and biscuits	0.05	0.01	0.46
Milk pudding	0.12	0.13	0.29
Fruits	−0.12	0.22	0.13
Eggs	−0.12	0.22	0.13
Milk	0.22	−0.04	0.28
Sugar-sweetened beverages	0.26	0.02	0.19
Alcohol	0.05	0.09	−0.36
Butter	0.36	−0.06	0.14
Margarine	−0.18	0.11	−0.10
Lard	0.33	0.04	0.01
Oil	0.04	0.09	−0.04
Cheese	0.10	0.09	−0.02
Cream	0.05	−0.04	0.34
Variance of food intake explained (%)	10.29	8.40	6.12

^1^ Component loadings, expressed as correlation coefficients (r), represent the magnitude and directions of the association with the components (dietary patterns) and ranges from −1.0 to 1.0; absolute factor loadings >0.20 were considered as the cut-off point for interpreting the three dietary patterns [28,29,30]; ^2^ The number of retained components was based on graphical evaluation of the scree plot.

**Table 2:** Descriptive characteristics of the 1838 middle-aged men in the CaPS at phase 2 ^1^.

Descriptive Characteristics	Phase 2	Phase 5	*p*-Value ^17^
Follow-up, years	16.6 ± 7.2	-	
Age, years	56.7 ± 4.5	72.8 ± 4.1 ^13^	
Current smoking, *n* (%)	791 (43.0%)	-	
Non-Manual worker, *n* (%)	620 (33.7%)	-	
Physically active ^2^, *n* (%)	810 (44.1%)	-	
Body mass index, kg/m^2^	26.3 ± 3.6 ^7^	-	
Systolic blood pressure, mmHg	145.5 ± 22.1 ^8^	141.3 ± 19.7 ^14^	0.294
Diastolic blood pressure, mmHg	84.5 ± 11.7 ^9^	74.4 ± 11.1 ^14^	<0.001
Augmentation Index, %	-	26.6 ± 9.3 ^14^	
Pulse wave velocity, m/s	-	11.2 (9.5–12.9) ^15^	
Total cholesterol, mmol/L	5.6 ± 1.0 ^10^	-	
LDL cholesterol, mmol/L	4.2 ± 0.9 ^10^	-	
HDL cholesterol, mmol/L	1.0 ± 0.2 ^10^	-	
Total:HDL cholesterol	5.8 ± 1.7 ^10^		
Triacylglycerol, mmol/L	1.6 (1.2–2.3) ^10^	1.4 (1.0–1.9) ^15^	<0.001
Glucose, mmol/L	5.2 ± 0.8 ^10^	-	
C-Reactive Protein, mg/L	1.6 (0.8–3.2) ^11^	2.9 (1.5–5.6) ^16^	<0.001
Framingham Risk Score ^3^, %	24.5 (16.9–34.2) ^12^	-	
Dietary intake ^4^			
Total energy intake, kcal/day	1989.8 ± 529.0	-	
Fat, g/day (%TE)	79.0 ± 24.1 (35.7)	-	
Saturated fatty acids, g/day (%TE)	36.4 ± 12.2 (16.5)		
Carbohydrates, g/day (%TE)	238.0 ± 70.4 (47.8)	-	
Total sugar, g/day (%TE)	90.3 ± 43.1 (18.2)		
Protein, g/day (%TE)	69.7 ± 16.6 (12.0)	-	
Fibre, g/day	20.6 ± 6.5	-	
Sodium ^5^, mg/day	2327.0 ± 615.8	-	
Cholesterol, mg/day	340.6 ± 101.5	-	
Vegetable intake, g/day	96.5 (72.4–130.1)	-	
Fruit intake, g/day	44.9 (16.5–91.4)	-	
Dairy intake ^6^, g/day	167.7 (125.4–295.8)	-	
Meat intake, g/day	96.7 (70.5–123)	-	
Fish intake, g/day	30.4 (19.6–42.9)	-	
Ethanol intake, g/day	10.8 (2.2–22.7)	-	

^1^ Data are presented as %, mean ± SD or as median with interquartile range when the variable was not normally distributed. %TE, percentage of total energy; ^2^ Physically active is estimated as having a total time spent on leisure activities higher than 2100 kcal/week; ^3^ Framingham Risk Score (FRS) for global CVD risk is calculated based on the formula including the risk factors age, systolic blood pressure not treated, total and HDL cholesterol, smoking status and diabetes; ^4^ Dietary intake is calculated as mean phase 2 and phase 3 dietary intake; ^5^ Sodium intake only from foods, not including discretionary salt; ^6^ Dairy intake, not including butter; ^7^ Data are available for 1814 men; ^8^ Data are available for 1819 men; ^9^ Data are available for 1818 men; ^1^^0^ Data are available for 1775 men; ^11^ Data are available for 1222 men; ^12^ Data are available for 1757 men; ^13^ Data are available for 760 men; ^14^ Data are available for 758 men; ^15^ Data are available for 730 men; ^16^ Data are available for 711 men; ^17^ *p*-values calculated with mixed models adjusted for age, smoking status, social class, physical activity level, energy intake and alcohol consumption.

**Table 3:** Associations between dietary patterns and incidence of cardiovascular disease, coronary heart disease and stroke in the Caerphilly Prospective Study ^1^.

Tertile	Cardiovascular Disease			Coronary Heart Disease			Stroke		
	Events	Crude HR (95% CI)	Adjusted HR ^2^ (95% CI)	Events	Crude HR (95% CI)	Adjusted HR ^2^ (95% CI)	Events	Crude HR (95% CI)	Adjusted HR ^2^ (95% CI)
Dietary pattern 1									
T1 (*n* = 612)	233	Reference	Reference	136	Reference	Reference	60	Reference	Reference
T2 (*n* = 613)	233	1.04 (0.87, 1.25)	1.02 (0.85, 1.24)	128	0.97 (0.76, 1.24)	0.93 (0.73, 1.20)	66	1.16 (0.82, 1.65)	1.17 (0.82, 1.68)
T3 (*n* = 612)	249	1.37 (1.15, 1.64)	1.35 (1.10, 1.67)	138	1.29 (1.02, 1.63)	1.23 (0.93, 1.63)	79	1.73 (1.23, 2.42)	1.77 (1.18, 2.63)
Dietary pattern 2									
T1 (*n* = 612)	234	Reference	Reference	144	Reference	Reference	60	Reference	Reference
T2 (*n* = 613)	242	1.03 (0.86, 1.24)	1.01 (0.84, 1.21)	140	0.97 (0.77, 1.22)	0.96 (0.76, 1.22)	68	1.15 (0.82, 1.63)	1.08 (0.76, 1.53)
T3 (*n* = 613)	239	1.06 (0.89, 1.27)	1.12 (0.93, 1.35)	118	0.85 (0.67, 1.08)	0.93 (0.72, 1.19)	77	1.36 (0.97, 1.90)	1.32 (0.93, 1.89)
Dietary pattern 3									
T1 (*n* = 612)	248	Reference	Reference	139	Reference	Reference	78	Reference	Reference
T2 (*n* = 613)	245	0.87 (0.73, 1.04)	0.83 (0.69, 1.00)	148	0.95 (0.75, 1.20)	0.87 (0.68, 1.11)	58	0.65 (0.46, 0.91)	0.62 (0.43, 0.88)
T3 (*n* = 613)	222	0.79 (0.66, 0.95)	0.76 (0.63, 0.93)	115	0.74 (0.58, 0.95)	0.68 (0.52, 0.90)	69	0.78 (0.56, 1.08)	0.68 (0.47, 0.99)

^1^ Values are hazard ratios (95% confidence intervals), *n* = 1838; ^2^ Adjusted for age, smoking habits, social class, leisure time physical activity, total energy intake and usual alcohol consumption.

**Table 4:** Longitudinal relationships between dietary patterns and cardiovascular risk markers in the Caerphilly Prospective Study ^1^.

Model	Dietary Pattern 1			Dietary Pattern 2			Dietary Pattern 3		
	T1	T2	T3	T1	T2	T3	T1	T2	T3
Systolic blood pressure, mmHg									
Crude	Reference	−1.28 (−4.55, 1.99)	−1.63 (−5.21, 1.94)	Reference	0.78 (−2.63, 4.20)	1.48 (−1.96, 4.91)	Reference	0.21 (−3.34, 3.76)	−0.82 (−4.36, 2.73)
Adjusted	Reference	−1.36 (−4.81, 2.09)	−1.93 (−6.21, 2.34)	Reference	−0.19 (−3.66, 3.28)	0.67 (−2.98, 4.33)	Reference	1.08 (−2.62, 4.77)	0.61 (−3.44, 4.67)
Diastolic blood pressure, mmHg									
Crude	Reference	1.04 (−0.80, 2.89)	1.03 (−0.99, 3.05)	Reference	−0.21 (−2.14, 1.71)	−0.36 (−2.30, 1.58)	Reference	0.06 (−1.94, 2.06)	−0.93 (−2.93, 1.07)
Adjusted	Reference	0.65 (−1.29, 2.59)	0.53 (−1.87, 2.93)	Reference	−0.53 (−2.47, 1.42)	−1.21 (−3.26, 0.84)	Reference	0.46 (−1.62, 2.53)	−0.64 (−2.91, 1.64)
Augmentation index, %									
Crude	Reference	−1.29 (−2.83, 0.24)	0.63 (−1.05, 2.31)	Reference	−0.09 (−1.70, 1.51)	1.52 (−0.10, 3.13)	Reference	0.90 (−0.77, 2.57)	0.09 (−1.57, 1.76)
Adjusted	Reference	−1.50 (−3.13, 0.12)	0.23 (−1.78, 2.24)	Reference	−0.07 (−1.70, 1.57)	1.74 (0.02, 3.46)	Reference	0.31 (−1.44, 2.05)	−0.93 (−2.85, 0.99)
Log aortic pulse wave velocity, m/s									
Crude	Reference	0.01 (−0.03, 0.06)	0.003 (−0.04, 0.05)	Reference	0.01 (−0.03, 0.05)	−0.02 (−0.06, 0.02)	Reference	−0.05 (−0.09, 0.00)	−0.05 (−0.09, 0.00)
Adjusted	Reference	0.03 (−0.01, 0.06)	0.03 (−0.02, 0.07)	Reference	−0.002(−0.04, 0.04)	−0.03 (−0.07, 0.01)	Reference	−0.03 (−0.07, 0.01)	−0.03 (−0.08, 0.02)
Log Triacylglycerol, mmol/L									
Crude	Reference	0.001 (−0.08, 0.08)	0.07 (−0.01, 0.16)	Reference	0.05 (−0.03, 0.14)	−0.03 (−0.11, 0.06)	Reference	0.001(−0.09, 0.09)	−0.09 (−0.18, 0.01)
Adjusted	Reference	−0.03 (−0.12, 0.05)	0.03 (−0.07, 0.14)	Reference	0.04 (−0.05, 0.12)	−0.05 (−0.14, 0.04)	Reference	0.03 (−0.06, 0.12)	−0.06 (−0.16, 0.04)
Log C-Reactive Protein, mg/L									
Crude	Reference	0.30 (0.09, 0.52)	0.08 (−0.15, 0.32)	Reference	0.16 (−0.06, 0.39)	0.06 (−0.17, 0.28)	Reference	−0.04 (−0.27, 0.19)	−0.31 (−0.54,−0.08)
Adjusted	Reference	0.26 (0.04, 0.49)	0.03 (−0.24, 0.31)	Reference	0.10 (−0.12, 0.33)	0.07 (−0.17, 0.31)	Reference	0.01 (−0.23, 0.26)	−0.18 (−0.45, 0.09)

^1^ Values are the crude and adjusted change in risk markers (95% CI). Adjusted for age, smoking status, social class, leisure time physical activity, total energy intake, usual alcohol consumption and BMI.

## Supplementary Materials

The following are available online at http://www.mdpi.com/2072-6643/9/1/075/s1, Table S1: Descriptive characteristics per tertile of component scores for dietary pattern 1 of the 1838 middle-aged men in the CaPS at phase 2, Table S2: Descriptive characteristics per tertile of component scores for dietary pattern 2 of the 1838 middle-aged men in the CaPS at phase 2, Table S3: Descriptive characteristics per tertile of component scores for dietary pattern 3 of the 1838 middle-aged men in the CaPS at phase 2, Table S4: Cross-sectional relationship between dietary patterns and cardiovascular risk markers in the Caerphilly Prospective Study.
