# Supplementary material for: Dietary Patterns in Relation to Cardiovascular Disease Incidence and Risk Markers in a Middle-Aged British Male Population: Data from the Caerphilly Prospective Study
Source: Nutrients. 2017 Jan 18;9(1):75. doi: 10.3390/nu9010075 (PMC5295119; doi:10.3390/nu9010075)
Supplement: Supplementary file 1 [file nutrients-09-00075-s001.docx]

Supplementary Materials: Dietary Patterns in Relation to Cardiovascular Disease Incidence and Risk Markers in a Middle-Aged British Male Population: Data from the Caerphilly Prospective Study

Elly Mertens, Oonagh Markey, Johanna M. Geleijnse, David. Ian Givens and Julie A. Lovegrove

**Table S1.** Descriptive characteristics per tertile of component scores for dietary pattern 1 of the 1838 middle-aged men in the CaPS at phase 2 ^1^.

| **Descriptive Characteristics** | **Tertiles of Component Scores** | | |
| --- | --- | --- | --- |
|  | **T1 (*n* = 612)** | **T2 (*n* = 613)** | **T3 (*n* = 613)** |
| Median factors | −1.69 (−2.32, −1.16) | −0.03 (−0.37, 0.34) | 1.60 (1.14, 2.25) |
| Follow-up, years | 17.5 ± 6.8 | 17.3 ± 7.0 | 15.1 ± 7.4 |
| Age at phase 2, years | 57.0 ± 4.4 | 56.5 ± 4.4 | 56.4 ± 4.7 |
| Current smoking, *n* (%) | 162 (26.5%) | 261 (42.6%) | 368 (60.0%) |
| Non-Manual worker, *n* (%) | 269 (44.0%) | 211 (34.4%) | 140 (22.8%) |
| Physically active, *n* (%) | 275 (44.9%) | 281 (45.4%) | 261 (42.6%) |
| Body mass index, kg/m^2^ | 26.8 ± 3.4 ^2^ | 26.7 ± 3.6 ^8^ | 25.6 ± 3.6 ^13^ |
| Systolic blood pressure, mmHg | 145.8 ± 21.5 ^3^ | 146.2 ± 22.8 ^9^ | 144.5 ± 22.2 ^14^ |
| Diastolic blood pressure, mmHg | 84.4 ± 11.4 ^3^ | 85.1 ± 11.8 ^10^ | 84.1 ± 11.8 ^15^ |
| Total cholesterol, mmol/L | 5.6 ± 1.0 ^4^ | 5.6 ± 1.0 ^11^ | 5.6 ± 1.0 ^16^ |
| LDL cholesterol, mmol/L | 4.2 ± 1.0 ^4^ | 4.2 ± 0.9 ^11^ | 4.2 ± 0.9 ^16^ |
| HDL cholesterol, mmol/L | 1.0 ± 0.2 ^4^ | 1.0 ± 0.2 ^11^ | 1.0 ± 0.3 ^16^ |
| Triacylglycerol, mmol/L | 1.6 (1.2–2.1) ^4^ | 1.6 (1.2–2.3) ^11^ | 1.7 (1.1–2.3) ^16^ |
| Glucose, mmol/L | 5.2 ± 0.7 ^5^ | 5.2 ± 0.7 ^11^ | 5.2 ± 1.0 ^17^ |
| C-Reactive Protein, mg/L | 1.4 (0.6–2.8) ^6^ | 1.6 (0.9–3.4) ^12^ | 1.7 (0.9–3.4) ^18^ |
| Framingham Risk Score, % | 22.5 (16.0–31.7) ^7^ | 24.5 (16.6–34.2) ^13^ | 26.4 (18.4–37.2) ^19^ |
| Dietary intake | | | |
| Total energy intake, kcal/day | 1736.0 ± 408.7 | 1939.5 ± 447.6 | 2293.6 ± 559.9 |
| Fat, g/day (%TE) | 66.9 ± 19.5 (34.7) | 76.9 ± 20.2 (35.7) | 93.1 ± 24.7 (36.5) |
| Saturated fatty acids, g/day (%TE) | 28.1 ± 8.2 (14.6) | 35.1 ± 8.3 (16.3) | 45.8 ± 11.5 (18.0) |
| Carbohydrates, g/day (%TE) | 207.9 ± 52.7 (47.9) | 229.4 ± 60.6 (47.3) | 276.5 ± 77.3 (48.2) |
| Total sugar, g/day (%TE) | 70.6 ± 26.7 (16.3) | 87.2 ± 33.8 (18.0) | 116.3 ± 50.0 (20.3) |
| Protein, g/day (%TE) | 65.8 ± 14.7 (15.2) | 68.2 ± 15.3 (14.1) | 75.0 ± 18.1 (13.1) |
| Fibre, g/day | 24.8 ± 6.8 | 19.0 ± 5.5 | 18.1 ± 4.8 |
| Sodium from foods, mg/day | 2151.5 ± 582.7 | 2263.6 ± 585.6 | 2566.1 ± 603.6 |
| Cholesterol, mg/day | 290.4 ± 75.3 | 332.1 ± 87.8 | 399.2 ± 107.3 |
| Vegetable intake, g/day | 119.9 (89.8–151.2) | 95.4 (72.7–123.7) | 83.9 (61.1–112.9) |
| Fruit intake, g/day | 66.8 (31.3–117.3) | 43.1 (16.4–84.8) | 32.6 (9.6–70.8) |
| Dairy intake, g/day | 133.9 (93.6–200.8) | 168.1 (129.8–299.8) | 241.8 (140.9–348.5) |
| Meat intake, g/day | 86.1 (61.4–110.1) | 94.3 (71.1–120.6) | 112.2 (84.3–139.4) |
| Fish intake, g/day | 34.8 (21.4–51.8) | 30.4 (19.6–41.1) | 30.4 (19.6–40.2) |
| Ethanol intake, g/day | 8.1 (1.7–17.8) | 12.0 (2.5–24.6) | 12.6 (2.6–25.3) |

^1^ Data are presented as %, mean ± SD or as median with interquartile range when the variable was not normally distributed. %TE, percentage of total energy; ^2^ Data are available for 608 men; ^3^ Data are available for 606 men; ^4^ Data are available for 594 men; ^5^ Data are available for 597 men; ^6^ Data are available for 423 men; ^7^ Data are available for 588 men; ^8^ Data are available for 601 men; ^9^ Data are available for 605 men; ^10^ Data are available for 604 men; ^11^ Data are available for 593 men; ^12^ Data are available for 405 men; ^13^ Data are available for 586 men; ^14^ Data are available for 605 men; ^15^ Data are available for 608 men; ^16^ Data are available for 588 men; ^17^ Data are available for 585 men; ^18^ Data are available for 394 men; ^19^ Data are available for 583 men.

**Table S2.** Descriptive characteristics per tertile of component scores for dietary pattern 2 of the 1838 middle-aged men in the CaPS at phase 2 ^1^.

| **Descriptive Characteristics** | **Tertiles of Component Scores** | | |
| --- | --- | --- | --- |
|  | **T1 (*n* = 612)** | **T2 (*n* = 613)** | **T3 (*n* = 613)** |
| Median factors | −1.37 (−1.81, −1.05) | −0.38 (−1.22, 0.59) | 1.31 (0.82, 2.08) |
| Follow-up, years | 16.5 ± 7.5 | 16.7 ± 7.0 | 16.6 ± 7.0 |
| Age at phase 2, years | 56.8 ± 4.6 | 56.7 ± 4.5 | 56.5 ± 4.4 |
| Current smoking, *n* (%) | 260 (42.5%) | 270 (44.0%) | 261 (42.6%) |
| Non-Manual worker, *n* (%) | 218 (35.6%) | 216 (35.2%) | 186 (30.3%) |
| Physically active, *n* (%) | 238 (38.9%) | 254 (41.4%) | 318 (51.9%) |
| Body mass index, kg/m^2^ | 26.2 ± 3.4 ^2^ | 26.3 ± 3.5 ^8^ | 26.5 ± 3.8 ^14^ |
| Systolic blood pressure, mmHg | 145.7 ± 22.0 ^3^ | 144.9 ± 22.8 ^9^ | 145.9 ± 21.6 ^15^ |
| Diastolic blood pressure, mmHg | 84.4 ± 11.6 ^3^ | 84.7 ± 11.8 ^10^ | 84.5 ± 11.7 ^15^ |
| Total cholesterol, mmol/L | 5.6 ± 1.0 ^4^ | 5.6 ± 1.0 ^11^ | 5.7 ± 0.9 ^16^ |
| LDL cholesterol, mmol/L | 4.2 ± 0.9 ^4^ | 4.2 ± 1.0 ^11^ | 4.2 ± 0.9 ^16^ |
| HDL cholesterol, mmol/L | 1.0 ± 0.2 ^4^ | 1.0 ± 0.2 ^11^ | 1.0 ± 0.3 ^16^ |
| Triacylglycerol, mmol/L | 1.6 (1.2–2.2) ^4^ | 1.6 (1.2–2.3) ^11^ | 1.6 (1.1–2.4) ^16^ |
| Glucose, mmol/L | 5.2 ± 0.7 ^5^ | 5.2 ± 0.8 ^11^ | 5.2 ± 0.8 ^17^ |
| C-Reactive Protein, mg/L | 1.5 (0.8–3.4) ^6^ | 1.6 (0.9–3.4) ^12^ | 1.5 (0.8–2.8) ^18^ |
| Framingham Risk Score, % | 24.4 (16.5–34.3) ^7^ | 24.2 (17.2–33.8) ^13^ | 24.7 (16.9–34.6) ^7^ |
| Dietary intake | | | |
| Total energy intake, kcal/day | 1864.9 ± 454.7 | 1954.9 ± 480.5 | 2149.4 ± 600.2 |
| Fat, g/day (%TE) | 75.2 ± 21.3 (36.3) | 77.4 ± 22.0 (35.6) | 84.4 ± 27.8 (35.3) |
| Saturated fatty acids, g/day (%TE) | 35.5 ± 10.9 (17.1) | 35.2 ± 10.9 (16.2) | 38.2 ± 13.6 (16.0) |
| Carbohydrates, g/day (%TE) | 225.5 ± 63.2 (48.4) | 232.9 ± 64.2 (47.7) | 255.5 ± 79.2 (47.5) |
| Total sugar, g/day (%TE) | 87.7 ± 41.1 (18.8) | 88.9 ± 40.7 (18.2) | 97.6 ± 45.0 (18.2) |
| Protein, g/day (%TE) | 61.9 ± 13.3 (13.3) | 68.4 ± 13.7 (14.0) | 78.7 ± 17.8 (14.6) |
| Fibre, g/day | 17.5 ± 4.9 | 20.4 ± 5.8 | 24.0 ± 6.9 |
| Sodium from foods, mg/day | 2094.6 ± 509.6 | 2259.7 ± 518.9 | 2626.9 ± 679.2 |
| Cholesterol, mg/day | 229.7 ± 76.7 | 329.1 ± 80.3 | 392.9 ± 118.5 |
| Vegetable intake, g/day | 79.8 (57.6–102.0) | 95.4 (72.7–123.7) | 122.8 (90.4–153.3) |
| Fruit intake, g/day | 27.6 (8.9–57.6) | 43.1 (16.4–84.6) | 72.3 (33.4–127.2) |
| Dairy intake, g/day | 181.0 (125.4–296.7) | 157.6 (124.6–291.1) | 163.4 (125.6–307.4) |
| Meat intake, g/day | 70.8 (53.2–89.7) | 94.3 (71.1–120.6) | 123.4 (101.1–149.6) |
| Fish intake, g/day | 21.4 (10.7–30.4) | 30.4 (21.4–41.1) | 43.8 (30.4–60.7) |
| Ethanol intake, g/day | 9.5 (1.5–19.0) | 12.0 (2.5–24.6) | 11.4 (2.9–24.6) |

^1^ Data are presented as %, mean ± SD or as median with interquartile range when the variable was not normally distributed. %TE, percentage of total energy; ^2^ Data are available for 604 men; ^3^ Data are available for 608 men; ^4^ Data are available for 588 men; ^5^ Data are available for 587 men; ^6^ Data are available for 403 men; ^7^ Data are available for 584 men; ^8^ Data are available for 602 men; ^9^ Data are available for 605 men; ^10^ Data are available for 604 men; ^11^ Data are available for 597men; ^12^ Data are available for 411 men; ^13^ Data are available for 589 men; ^14^ Data are available for 608 men; ^15^ Data are available for 606 men; ^16^ Data are available for 590 men; ^17^ Data are available for 591 men; ^18^ Data are available for 408 men.

**Table S3.** Descriptive characteristics per tertile of component scores for dietary pattern 3 of the 1838 middle-aged men in the CaPS at phase 2 ^1^.

| **Descriptive Characteristics** | **Tertiles of Component Scores** | | |
| --- | --- | --- | --- |
|  | **T1 (*n* = 612)** | **T2 (*n* = 613)** | **T3 (*n* = 613)** |
| Median factors | −1.29 (−1.71, −0.94) | −0.11 (−0.40, 0.18) | 1.30 (0.83, 1.92) |
| Follow-up, years | 15.8 ± 7.4 | 17.2 ± 7.0 | 16.9 ± 7.0 |
| Age at phase 2, years | 56.3 ± 4.5 | 56.7 ± 4.4 | 56.8 ± 4.4 |
| Current smoking, *n* (%) | 308 (50.3%) | 270 (44.0%) | 213 (34.7%) |
| Non-Manual worker, *n* (%) | 160 (26.1%) | 206 (33.6%) | 254 (41.4%) |
| Physically active, *n* (%) | 268 (43.8%) | 259 (42.3%) | 283 (46.2%) |
| Body mass index, kg/m^2^ | 26.8 ± 3.7 ^2^ | 26.4 ± 3.6 ^8^ | 25.8 ± 3.3 ^14^ |
| Systolic blood pressure, mmHg | 147.8 ± 22.8 ^3^ | 144.4 ± 22.4 ^9^ | 144.3 ± 21.0 ^15^ |
| Diastolic blood pressure, mmHg | 85.4 ± 11.9 ^3^ | 84.3 ± 11.8 ^9^ | 83.9 ± 11.3 ^16^ |
| Total cholesterol, mmol/L | 5.7 ± 1.0 ^4^ | 5.6 ± 0.9 ^10^ | 6.0 ± 1.0 ^17^ |
| LDL cholesterol, mmol/L | 4.2 ± 1.0 ^4^ | 4.2 ± 0.9 ^10^ | 4.2 ± 0.9 ^17^ |
| HDL cholesterol, mmol/L | 1.0 ± 0.3 ^4^ | 1.0 ± 0.2 ^10^ | 1.0 ± 0.2 ^17^ |
| Triacylglycerol, mmol/L | 1.8 (1.3–2.6) ^4^ | 1.6 (1.2–2.2) ^10^ | 1.5 (1.1–2.0) ^17^ |
| Glucose, mmol/L | 5.2 ± 0.7 ^5^ | 5.2 ± 0.7 ^11^ | 5.2 ± 0.9 ^18^ |
| C-Reactive Protein, mg/L | 1.8 (0.9–3.5) ^6^ | 1.6 (0.8–3.2) ^12^ | 1.3 (0.6–2.7) ^19^ |
| Framingham Risk Score, % | 25.1 (17.4–35.4) ^7^ | 24.2 (16.5 -34.8) ^13^ | 23.9 (17.0–32.8) ^20^ |
| Dietary intake | | | |
| Total energy intake, kcal/day | 1959.3 ± 548.0 | 1937.7 ± 502.8 | 2072.3 ± 526.1 |
| Fat, g/day (%TE) | 75.4 ± 24.3 (34.6) | 77.0 ± 22.6 (35.8) | 84.6 ± 24.5 (36.7) |
| Saturated fatty acids, g/day (%TE) | 33.9 ± 11.3 (15.6) | 34.8 ± 11.1 (16.2) | 40.2 ± 12.5 (17.5) |
| Carbohydrates, g/day (%TE) | 221.2 ± 67.0 (45.2) | 234.7 ± 67.8 (48.4) | 258.1 ± 71.4 (49.8) |
| Total sugar, g/day (%TE) | 83.5 ± 39.2 (17.0) | 87.0 ± 41.8 (18.0) | 103.6 ± 43.8 (20.0) |
| Protein, g/day (%TE) | 66.6 ± 17.0 (13.6) | 68.9 ± 15.3 (14.2) | 73.5 ± 16.6 (14.2) |
| Fibre, g/day | 17.5 ± 5.4 | 20.9 ± 6.0 | 23.5 ± 6.4 |
| Sodium from foods, mg/day | 2281.1 ± 630.1 | 2289.4 ± 600.3 | 2410.6 ± 609.0 |
| Cholesterol, mg/day | 334.6 ± 102.8 | 329.2 ± 98.8 | 357.8 ± 100.7 |
| Vegetable intake, g/day | 82.3 (56.9–108.2) | 100.5 (74.3–130.4) | 116 (85.1–147.1) |
| Fruit intake, g/day | 31.9 (8.6–68.0) | 46.8 (20.4–92.1) | 63.0 (25.1–110.1) |
| Dairy intake, g/day | 133.9 (107.3–190.7) | 162.7 (126.0–281.5) | 272.9 (151.4–354.5) |
| Meat intake, g/day | 99.3 (71.0–125.3) | 94.1 (67.6–120.6) | 97.1 (71.6–122.8) |
| Fish intake, g/day | 30.4 (19.6–42.6) | 30.4 (19.6–42.9) | 32.1 (21.4–46.4) |
| Ethanol intake, g/day | 20.2 (10.1–41.7) | 10.1 (2.2–20.2) | 4.5 (0.9–12.2) |

^1^ Data are presented as %, mean ± SD or as median with interquartile range when the variable was not normally distributed. %TE, percentage of total energy; ^2^ Data are available for 606 men; ^3^ Data are available for 604 men; ^4^ Data are available for 590 men; ^5^ Data are available for 594 men; ^6^ Data are available for 408 men; ^7^ Data are available for 583 men; ^8^ Data are available for 604 men; ^9^ Data are available for 608 men; ^10^ Data are available for 590 men; ^11^ Data are available for 588 men; ^12^ Data are available for 392 men; ^13^ Data are available for 585 men; ^14^ Data are available for 604 men; ^15^ Data are available for 607 men; ^16^ Data are available for 606 men; ^17^ Data are available for 595 men; ^18^ Data are available for 593 men; ^19^ Data are available for 422 men; ^20^ Data are available for 589 men.

**Table S4.** Cross-sectional relationship between dietary patterns and cardiovascular risk markers in the Caerphilly Prospective Study ^1^.

| **Model** | **Dietary Pattern 1** | | | **Dietary Pattern 2** | | | **Dietary Pattern 3** | | |
| --- | --- | --- | --- | --- | --- | --- | --- | --- | --- |
|  | **Tertiles of Component Scores** | | | **Tertiles of Component Scores** | | | **Tertiles of Component Scores** | | |
|  | **T1** | **T2** | **T3** | **T1** | **T2** | **T3** | **T1** | **T2** | **T3** |
| Body Mass Index, kg/m ^2^ | | | | | | | | | |
| Crude | Reference | −0.18 (−0.57, 0.22) | −1.19 (−1.59, −0.80) | Reference | 0.16 (−0.24, 0.56) | 0.33 (−0.07, 0.73) | Reference | −0.44 (−0.84, −0.04) | −1.05 (−1.45, −0.65) |
| Adjusted | Reference | 0.03 (−0.36, 0.43) | −0.58 (−1.03, −0.13) | Reference | 0.22 (−0.17, 0.60) | 0.52 (0.12, 0.92) | Reference | −0.47 (−0.87, −0.06) | −0.99 (−1.43, −0.55) |
| Systolic blood pressure, mmHg | | | | | | | | | |
| Crude | Reference | 0.21 (−2.02, 2.44) | −0.39 (−2.62, 1.84) | Reference | −0.64 (−2.87, 1.59) | 0.78 (−1.45, 3.01) | Reference | −3.75 (−5.97, −1.53) | −4.61 (−6.83, −2.39) |
| Adjusted | Reference | 0.40 (−1.80, 2.61) | 0.72 (−1.78, 3.21) | Reference | −1.00 (−3.14, 1.15) | 0.44 (−1.77, 2.64) | Reference | −2.42 (−4.64, −0.20) | −3.07 (−5.50, −0.64) |
| Diastolic blood pressure, mmHg | | | | | | | | | |
| Crude | Reference | 0.54 (−0.61, 1.69) | 0.13 (−1.02, 1.29) | Reference | 0.08 (−1.08, 1.23) | 0.69 (−0.46, 1.85) | Reference | −0.83 (−1.99, 0.32) | −1.42 (−2.57, −0.27) |
| Adjusted | Reference | 0.86 (−0.28, 2.01) | 1.57 (0.26, 2.85) | Reference | −0.03 (−1.14, 1.09) | 0.30 (−0.85, 1.45) | Reference | −0.43 (−1.58, 0.73) | −0.68 (−1.94, 0.59) |
| Total cholesterol, mmol/L | | | | | | | | | |
| Crude | Reference | 0.02 (−0.09, 0.13) | 0.04 (−0.07, 0.15) | Reference | 0.02 (−0.09, 0.13) | 0.004 (−0.11, 0.12) | Reference | −0.01 (−0.12, 0.10) | −0.04 (−0.16, 0.07) |
| Adjusted | Reference | 0.01 (−0.10, 0.13) | 0.03 (−0.10, 0.17) | Reference | 0.02 (−0.10, 0.13) | −0.01 (−0.13, 0.11) | Reference | 0.01 (−0.11, 0.13) | −0.04 (−0.17, 0.09) |
| LDL cholesterol, mmol/L | | | | | | | | | |
| Crude | Reference | −0.01 (−0.11, 0.10) | 0.01 (−0.09, 0.12) | Reference | −0.01 (−0.11, 0.10) | 0.01 (−0.09, 0.12) | Reference | 0.02 (−0.08, 0.13) | 0.03 (−0.08, 0.13) |
| Adjusted | Reference | 0.02 (−0.10, 0.12) | 0.07 (−0.06, 0.19) | Reference | 0.01 (−0.09, 0.12) | 0.03 (−0.07, 0.14) | Reference | 0.005 (−0.10, 0.11) | −0.02 (−0.14, 0.10) |
| HDL cholesterol, mmol/L | | | | | | | | | |
| Crude | Reference | 0.01 (−0.02, 0.04) | 0.01 (−0.02, 0.04) | Reference | 0.01 (−0.02, 0.04) | 0.02 (−0.01, 0.05) | Reference | −0.03 (−0.05, 0.003) | −0.02 (−0.05, 0.01) |
| Adjusted | Reference | 0.01 (−0.02, 0.04) | 0.004 (−0.03, 0.04) | Reference | 0.01 (−0.02, 0.04) | 0.01 (−0.02, 0.04) | Reference | −0.02 (−0.04, 0.01) | −0.01 (−0.05, 0.02) |
| Total:HDL cholesterol | | | | | | | | | |
| Crude | Reference | −0.04 (−0.24, 0.15) | −0.01 (−0.20, 0.19) | Reference | −0.08 (−0.27, 0.12) | −0.01 (−0.21, 0.18) | Reference | 0.05 (−0.14, 0.24) | −0.06 (−0.25, 0.13) |
| Adjusted | Reference | −0.07 (−0.27, 0.12) | 0.02 (−0.20, 0.24) | Reference | −0.04 (−0.23, 0.15) | 0.03 (−0.16, 0.23) | Reference | 0.03 (−0.17, 0.22) | −0.07 (−0.29, 0.15) |
| Log Triacylglycerol, mmol/L | | | | | | | | | |
| Crude | Reference | 0.04 (−0.01, 0.10) | 0.04 (−0.02, 0.09) | Reference | 0.04 (−0.01, 0.10) | 0.02 (−0.03, 0.08) | Reference | −0.07 (−0.12, −0.01) | −0.17 (−0.23, −0.12) |
| Adjusted | Reference | 0.02 (−0.04, 0.07) | 0.03 (−0.03, 0.09) | Reference | 0.03 (−0.02, 0.08) | 0.01 (−0.05, 0.06) | Reference | −0.02 (−0.08, 0.03) | −0.09 (−0.15, −0.03) |
| Blood glucose, mmol/L | | | | | | | | | |
| Crude | Reference | 0.02 (−0.06, 0.10) | 0.03 (−0.05, 0.12) | Reference | −0.01 (−0.09, 0.07) | 0.04 (−0.05, 0.12) | Reference | −0.03 (−0.11, 0.05) | −0.05 (−0.13, 0.04) |
| Adjusted | Reference | 0.07 (−0.01, 0.15) | 0.17 (0.08, 0.26) | Reference | −0.02 (−0.09, 0.06) | 0.07 (−0.01, 0.15) | Reference | −0.02 (−0.10, 0.06) | −0.03 (−0.12, 0.05) |
| Log C-Reactive Protein, mg/L | | | | | | | | | |
| Crude | Reference | 0.21 (0.08, 0.35) | 0.25 (0.11, 0.38) | Reference | −0.02 (−0.16, 0.12) | −0.09 (−0.23, 0.05) | Reference | −0.12 (−0.25,0.02) | −0.33 (−0.46, −0.19) |
| Adjusted | Reference | 0.11 (−0.02, 0.24) | 0.13 (−0.19, 0.28) | Reference | −0.03 (−0.16, 0.10) | −0.07 (−0.21, 0.06) | Reference | −0.05 (−0.18, 0.08) | −0.17 (−0.32, −0.03) |
| Log Framingham Risk Score ^2^ | | | | | | | | | |
| Crude | Reference | 0.06 (0.002, 0.12) | 0.13 (0.07, 0.19) | Reference | −0.001(−0.06, 0.06) | −0.003(−0.06, 0.06) | Reference | −0.05 (−0.11, 0.01) | −0.07 (−0.13, −0.01) |
| Adjusted | Reference | 0.06 (−0.003, 0.11) | 0.12 (0.06, 0.19) | Reference | 0.001 (−0.06, 0.06) | −0.01 (−0.07, 0.05) | Reference | −0.08 (−0.11, 0.01) | −0.08 (−0.14, −0.01) |

^1^ Values are the crude and adjusted change in risk markers (95% CI). Adjusted for age, smoking status, social class, leisure time physical activity, total energy intake and usual alcohol consumption; blood pressure, serum lipids, blood glucose and C-reactive protein levels were additionally adjusted for BMI; ^2^ No adjustment for age and smoking status in the multivariable model.
